# Dietary Supplementation of *Bacillus subtilis* Improves In Vitro Rumen Microbial Fermentation When Using *Macadamia integrifolia* Husk as a Substrate

**DOI:** 10.3390/microorganisms14071406

**Published:** 2026-06-25

**Authors:** Hu Liu, Xiaoyan Deng, Anmiao Chen, Hui Zeng, Qian Yang, Xingyu Chen, Kaibin Chen, Shiyang Huang, Xiaosong Zhang, Hanlin Zhou, Jiancheng Han

**Affiliations:** 1 Zhanjiang Experimental Station, Chinese Academy of Tropical Agricultural Sciences, Zhanjiang 524013, China; liuh2018@lzu.edu.cn (H.L.);; 2 College of Animal Science and Technology, Guangxi University, Nanning 530004, China; 3 Key Laboratory of Hainan Province for Postharvest Physiology and Technology of Tropical Horticultural Products, South Subtropical Crops Research Institute, Chinese Academy of Tropical Agricultural Sciences, Zhanjiang 524091, China; 4 College of Veterinary Medicine, China Agricultural University, Beijing 100193, China

**Keywords:** *Bacillus subtilis*, macadamia integrifolia husk, in vitro study, goats, rumen bacteria

## Abstract

Macadamia integrifolia husk (MIH) is a major byproduct of Macadamia integrifolia processing; however, there is limited information available on its rumen fermentation characteristics and associated bacterial communities when used as a feed for ruminants. This study evaluated the effects of different *Bacillus subtilis* (BS; ≥10^13^ CFU/g) inclusion levels on MIH using an in vitro technique. A total of six healthy goats (female, 7-month-old) with an average body weight of 15.20 ± 0.20 kg were selected as rumen fluid donors, and the rumen fluid was collected before morning feeding. Four BS inclusion levels were studied: a control group with 0 g/kg (CON), and three treatment groups supplemented with 1.5 g/kg (low dose, BSL), 3.0 g/kg (medium dose, BSM), and 4.5 g/kg (high dose, BSH) of BS. MIH supplemented with BS improved the gas production at all the observed incubation times (*p* < 0.05). The in vitro dry matter degradability was increased by 3.5%, 2.0%, and 3.1% in the BSL, BSM, and BSH groups, respectively, compared to the CON group at a 48 h incubation time (*p* = 0.049). The in vitro neutral detergent fiber degradability was increased by 6.0% and 7.8% in the BSL and BSM groups, respectively, compared to the CON group at a 48 h incubation time (*p* = 0.027). The in vitro acid detergent fiber degradability was increased by 11.7%, 16.8%, and 3.2% in the BSL, BSM, and BSH groups, respectively, compared with the CON group at the 48 h incubation time (*p* = 0.014). The concentrations of ammonia-N, microbial protein, total VFAs, acetate, propionate, butyrate, iso-butyrate, valerate, and iso-valerate were quadratically changed with increasing BS supplementation levels (*p* < 0.05). The relative abundances of Bacteroidota and Pseudomonadota were highest in the BSL group and lowest in the BSM group (*p* < 0.05), whereas Bacillota and Kiritimatiellota were highest in the BSH group and lowest in the BSL group (*p* < 0.05). Synergistota was highest in the BSM group and lowest in the BSH group (*p* < 0.05). The relative abundances of *norank_p_Bacteroidota* and *norank_o_Bacteroidoles* were highest in the BSL group, whereas they were lowest in the BSM group (*p* < 0.05). The relative abundances *Hoyesella* and *Succinivibrio* were highest in the BSL group, whereas they were lowest in the BSH group (*p* < 0.05). The relative abundances of *Prevotella*, *Succiniclasticum*, and *Selenomonas* were highest in the BSM group, whereas they were lowest in the BSL group (*p* < 0.05). These in vitro results indicate that supplementation with *Bacillus subtilis* could improve the utilization of MIH in goats, which is possibly associated with the altered rumen bacteria communities. The supplementation of 1.5 to 3.0 g/kg *Bacillus subtilis* (≥10^13^ CFU/g) on Macadamia integrifolia husk could improve its utilization as a feasible alternative feed for ruminants using an in vitro technique.

## 1. Introduction

*Macadamia integrifolia*, a member of the Proteaceae family, is an evergreen tree native to eastern Australia, prized for its nuts’ riches in essential amino acids and polyunsaturated fatty acids, which are beneficial to human health [1,2]. Currently, the global *Macadamia integrifolia* industry has expanded markedly and reached 344,240 metric tons (in-shell basis) in 2025 [3]. They are commercially cultivated in tropical and subtropical regions, with China emerging as the world’s largest and fastest-growing Macadamia producer. By 2025, China’s cultivated area exceeded 74,500 hectares [4,5].

The processing of *Macadamia integrifolia* nuts generates substantial quantities of lignocellulosic byproducts. Notably, the husk accounts for approximately 40% of the whole fruit weight [6]. Currently, the vast majority of these husks are discarded in landfills, with only a small fraction utilized by farmers as a rudimentary animal feed. This disposal method not only incurs a significant economic loss, but also poses environmental challenges related to waste management. With the growing global emphasis on agricultural sustainability and the circular bioeconomy, there is an urgent need to identify these underutilized byproduct residues for high-value applications. In the context of animal husbandry, repurposing *Macadamia integrifolia* husks (MIHs) as an alternative feedstuff for ruminants represents a promising strategy. However, the successful application of this agro-industrial residue requires a thorough investigation into its nutritional composition, palatability, and effects on animal growth performance [7,8,9]. Abulaiti et al. confirmed the feasibility of the functional utilization of walnut green husk as an unconventional feed resource for enhancing silage quality by driving beneficial microbial shifts. Therefore, an inclusion rate of 30 g·kg^−1^ was recommended as the most appropriate to achieve optimal in vitro fermentation and nutrient digestibility in finishing sheep [10]. In addition, the inclusion of pistachio husk in the diet of sheep caused an increase in the ruminal pH and reduced the concentrations of ammonia-N and volatile fatty acids in rumen liquor [11].

Fruit and vegetable peels are nutritionally and functionally valuable, and their use as feed in livestock could benefit both animal health and production performance [12]. In addition, additives—for example, formic acid, a mixture of acetic and lactic acids, or *Lentilactobacillus buchner*—and ensilage strategies could improve the utilization of almond hulls [9]. In our previous study, we found that supplementation with 1.5 g/kg cellulase and 0.5 g/kg pectinase in MIH could improve the in vitro disappearance of dry matter, neutral detergent fiber, and acid detergent fiber by altering the rumen bacterial community via an in vitro technique [13].

*Bacillus subtilis* (BS), as a ubiquitous bacterium, is widely used as a direct-fed microbial in livestock production. Maylem et al. reported that supplementation with 2 × 10^9^ CFU/head/day BS in lactating Holstein cows could increase feed efficiency and milk production by modulating the composition of ruminal microbiota and metabolism [14]. In addition, supplementing milk replacer with 0.2% BS effectively enhanced growth performance, maintained intestinal health, and altered the intestinal microbiota in Hu lambs [15]. Despite these benefits, the feasibility of BS supplementation with MIH has been scarcely investigated, and the doses for the supplementation levels are unclear.

To improve the utilization of MIH as a feedstuff in ruminants, we hypothesized that supplementation with BS could improve in vitro digestibility and alter rumen bacterial communities. Therefore, to test this hypothesis, we selected goats as the rumen fluid donors, and the objective of this study was to determine the effects of different levels of BS supplementation on in vitro gas production nutrient degradability, volatile fatty acid profiles, and bacterial community composition, using MIH as the substrate.

## 2. Materials and Methods

All animal care and experimental procedures followed the ethical guidelines approved by the Institutional Animal Care and Use Committee at the Zhanjiang Experimental Station, Chinese Academy of Tropical Agricultural Sciences (CATAS; protocol no. CATAS-20250001ZES; 6 January 2026).

### 2.1. Macadamia integrifolia Husk Sample and the Rumen Fluid Collection

*Macadamia integrifolia* husks were collected from Germplasm Resource Nursery, Institute of South Subtropical Crops, CATAS, located in Mazhang District, Zhanjiang City, Guangdong Province, China (21°09′52″ N and 110°16′24″ E). After collection, the *Macadamia integrifolia* husks were freeze-dried to a constant weight and ground using a hammer mill equipped with a 1 mm screen (HM-Lab, Guibao Group, Wuxi, China).

Rumen fluid (200 mL/goat) was collected by using an oral stomach tube before the morning feeding of six healthy Hainan black goats (female, 7 months old) with an average body weight of 15.20 ± 0.20 kg. The rumen fluid was filtered through four layers of cheesecloth, pooled, and mixed evenly. The mixed rumen fluid was immediately transferred into a pre-warmed thermos flask pre-flushed with CO_2_, maintained at 39 °C in an additional temperature-controlled container, and rapidly transported to the laboratory. The rumen fluid donors were fed a standardized diet (50% King grass, 36% cracked corn, 10% soybean meal, and 4% vitamin–mineral premix, DM basis) twice daily (0800 h and 1700 h) and had free access to feed and water. The diet provided 8.26 MJ/kg metabolizable energy and 110 g/kg crude protein to the goats.

### 2.2. Experimental Design and Ruminal Fermentation In Vitro

A total of four treatments were designed in this study, including one basal diet and three BS supplementation treatments. MIH served as the control (CON). In the experimental treatments, BS was supplemented at 1.5 g/kg (BSL group), 3.0 g/kg (BSM group), and 4.5 g/kg (BSH group) on a dry matter (DM) basis to the *Macadamia integrifolia* husk. The BS products were provided by STROWIN Co., Ltd. (≥10^13^ CFU/g; Beijing, China).

Approximately 400 mg of each substrate was weighed and placed into an individual nylon bag (4.5 cm × 5 cm; 38–40 μm, Bovine online, Beijing, China) along with 6.0 g of glass beads, ensuring that the bag remained submerged within the ruminal inoculum. The rumen fluid was combined and mixed at a ratio of 1:2 (*v*/*v*) with a buffer medium under CO_2_ flushing at 5 kpa, and the inoculum was prepared according to Liu et al. [16]. The buffer consisted of mineral solution, trace element solution, and buffer solution, and was prepared according to the method described by Menke et al. [17]. In vitro incubations were conducted using a 100 mL Menke fermenter (Model Fortuna, Haberle Labortechnik, Lonsee, Germany) placed in a shaking water bath maintained at 39 °C inside a biochemical incubator. A total of 70 Menke fermenters per run were used, and each contained one nylon bag and 40.0 mL of buffered rumen fluid. Each treatment consisted of four replicates per run at 6, 12, 24, and 48 h, respectively. A total of two runs were conducted in this experiment. Three standard oat hay samples (University of Hohenheim, Stuttgart, Germany) and three blanks (buffered rumen fluid only) were incubated concomitantly in each run for gas correction. All Menke fermenters were incubated at 39 °C in a biochemical incubator (SHP-205; Peiyin, Shanghai, China). Gas production (GP) was monitored and recorded at 3, 6, 9, 12, 24, and 48 h of incubation. Fermentation was stopped at 6, 12, 24, and 48 h of incubation, respectively. The nylon bags containing substrate residues were placed in ice water for 30 min to terminate microbial fermentation and then stored for subsequent analysis. After 48 h of incubation, culture solution samples were collected for the analysis of ruminal pH, volatile fatty acids (VFAs), ammonia-N, microbial protein, and the microbial community. A total of 5 mL of rumen fermentation sample was acidified with 1 mL of 25% (wt/vol) metaphosphoric acid before freezing for later VFA analysis.

### 2.3. The Chemical Composition of the Substrate and the Residuals Analysis

The *Macadamia integrifolia* husk substrate was analyzed for dry matter (DM, method 942.45), crude protein (CP, method 976.05), organic matter (OM, method 942.05), and ether extract (EE, method 920.29) according to the Association of Official Analytical Chemists [18]. Neutral detergent fiber (NDF) and acid detergent fiber (ADF) were determined using an automatic fiber analyzer (Ankom Technology, Fairport, NY, USA) according to Van Soest et al. [19] and Robertson and Van Soest [20], respectively. The substrate residues were also analyzed for DM, NDF, and ADF. The disappearance of DM, NDF, and ADF was calculated based on their initial contents in the substrate at 0 h and their residues after 6, 12, 24, and 48 h of fermentation.

### 2.4. Rumen Fermentation Parameter Analysis

The fermentation fluid samples were thawed at 4 °C and centrifuged at 3770× *g* for 15 min. Volatile fatty acid (VFA) concentrations, including acetate, propionate, butyrate, valerate, isobutyrate, and isovalerate, were determined using a gas chromatograph (SP-3420A; Beifen-Ruili Analytical Instrument Co., Ltd., Beijing, China) equipped with an AT-FFAP capillary column (30 m × 0.32 mm × 0.5 μm). The supernatant (1.5 mL) was transferred into a 2.0 mL tube containing 0.2 mL of 2-ethylbutyric acid as an internal standard. After vortexing and standing for 30 min, the mixture was centrifuged again at 3770× *g* for 15 min, and the supernatant was filtered (0.22 μm) prior to VFA determination. The injector temperature was set at 200 °C; the column temperature was increased from 45 to 130 °C at 10 °C/min and then held at 130 °C for 20 min. Ammonia-N and microbial protein (MCP) concentrations of the 48 h fermentation fluid were analyzed using a microplate reader according to the methods described by Hristov et al. [21] and Makkar et al. [22], respectively. For MCP analysis, fermentation rumen fluid samples (5 mL) were first centrifuged at 12,000 r/min for 20 min at 4 °C. After discarding the supernatant, the pellets were washed twice with distilled water. The washed pellets were brought to a final volume of 2 mL with distilled water and vortexed for 1 min. From this bacterial suspension, 1 mL was taken and mixed with an equal volume (1 mL) of 2 mol/L NaOH. The mixture was heated in a 95 °C water bath for 10 min and subsequently cooled. Following a second centrifugation step (10,000 r/min, 4 °C, 10 min), the supernatant was collected. A 1 mL portion of this supernatant was combined with 1.5 mL of 0.833 mol/L HCl following Makkar et al. [22].

### 2.5. Rumen Bacterial Communities’ Analysis

Microbial DNA was extracted from fermentation rumen fluid samples at 48 h of incubation using the E.Z.N.A.^®^ DNA Kit (Omega Bio-tek, Norcross, GA, USA) according to the manufacturer’s instructions. The DNA quality and concentration were determined using 1.0% agarose gel electrophoresis and a NanoDrop2000 spectrophotometer (Thermo Scientific, Wilmington, DE, USA), and the samples were kept at −80°C prior to further use. The hypervariable region of the bacterial 16S rRNA gene was amplified using the primer pair 27F (5′-AGRGTTYGATYMTGGCTCAG-3′) and 1492R (5′-RGYTACCTTGTTACGACTT-3′) in a T100 Thermal Cycler PCR thermocycler (BIO-RAD, Wilmington, DE, USA). The 20 µL PCR reaction mixture contained 10 μL 2 × Pro Taq, 0.8 μL forward primer (5 μM), 0.8 μL reverse primer (5 μM), 10 ng/μL of template DNA, and ddH_2_O to a final volume of 20 µL. The PCR thermal cycling protocol was as follows: initial denaturation at 95 °C for 3 min, followed by 27 cycles of denaturing at 95 °C for 30 s, annealing at 55 °C for 30 s and extension at 72 °C for 45 s, and single extension at 72 °C for 10 min, ending at 10 °C. The PCR product was extracted from 2% agarose gel, purified using the PCR Clean-Up Kit (YuHua, Shanghai, China) according to the manufacturer’s instructions, and quantified using Qubit 4.0 (Thermo Fisher Scientific, Waltham, MA, USA). Purified products were pooled in equimolar, and a DNA library was constructed using the SMRTbell prep kit 3.0 (Pacifc Biosciences, Menlo Park, CA, USA) according to PacBio’s instructions. Purified SMRTbell libraries were sequenced on the Pacbio Sequel IIe System (Pacifc Biosciences, Menlo Park, CA, USA) by Majorbio Bio-Pharm Technology Co. Ltd. (Shanghai, China). High-fidelity (HiFi) reads were obtained from the subreads, generated using circular consensus sequencing via SMRT Link v11.0. HiFi reads were barcode-identified and length-filtered. For bacterial 16S rRNA gene, sequences with a length <1000 or >1800 bp were removed. The taxonomy of each OTU representative sequence was analyzed by RDP Classifier version 2.2 against the 16S rRNA gene database (Silva v138) using a confidence threshold of 0.7. The data for microbial analysis used Majorbio Cloud (https://www.majorbio.com/; accessed on 2 April 2026).

### 2.6. Statistical Analysis

Statistical analyses for gas production, in vitro nutrient degradability, and rumen fermentation parameters were performed using the one-way ANOVA in GraphPad Prism (10.1.2; Beijing, China) with treatment as the fixed effect. To isolate the dosage effect, data were analyzed separately for each *Bacillus subtilis* treatment across the CON, 1.50 g/kg, 3.00 g/kg, and 4.50 g/kg inclusion levels.

Y_ij_ = u + T_i_ + e_ij_

where Y_ij_ is the observed value, μ is the overall mean, T_i_ is the fixed effect of treatment i (i  =  1, 2, 3), and e_ij_ is the residual error. Orthogonal polynomial contrasts were applied to evaluate the linear and quadratic effects of different BS supplementation groups on the gas production, in vitro nutrient degradability, and rumen fermentation parameters. When a significant overall treatment effect was detected, multiple pairwise comparisons were conducted using Tukey’s test.

## 3. Results

### 3.1. In Vitro Gas Production and In Vitro Nutrient Degradability

Compared to the CON group, the BS supplementation improved the gas production at the 3, 6, 9, 12, 24 and 48 h incubation times (*p* < 0.05; Table 1). Among the four groups, the gas production was highest in the BSL group at the 6 h incubation time and in the BSM group at the 3, 9, 12, 24, and 48 h incubation times.

The in vitro nutrient degradability was greater in the BS supplementation groups than in the CON group at the 6, 12, 24, and 48 h incubation times (*p* < 0.05; Table 2). Among the four groups, the DMD was highest in the BSM group and lowest in the CON group at the 6, 12, and 24 h incubation times (*p* < 0.05), whereas it was highest in the BSL group and lowest in the CON group at the 48 h incubation time (*p* < 0.05). The NDFD was highest in the BSM group, whereas it was lowest in the CON group at the 6, 12, 24, and 48 h incubation times (*p* < 0.05).

### 3.2. In Vitro Rumen Fermentation Parameters

There was no difference in in vitro ruminal pH among the four groups (*p* > 0.10; Table 3). The concentrations of ammonia-N, MCP, total VFAs, acetate, propionate, butyrate, iso-butyrate, valerate, and iso-valerate quadratically changed with increasing BS supplementation levels (*p* < 0.05). Among the four groups, the concentrations of ammonia-N, MCP, acetate, and iso-butyrate were highest in the BSL group (*p* < 0.05), and those of total VFAs, propionate, butyrate, valerate, and iso-valerate were highest in the BSM group (*p* < 0.05). The ratio of acetate to propionate decreased linearly with an increasing BS supplementation levels (*p* < 0.05).

### 3.3. In Vitro Rumen Bacterial Communities

**Figure 1:**
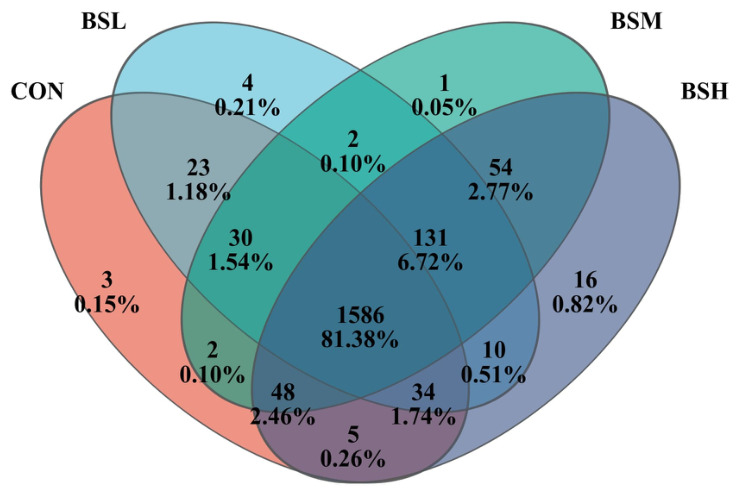
Venn diagram of the bacterial OTUs in rumen bacteria at 48 h incubation time across the four treatment groups (*n* = 8 per treatment). CON: no *Bacillus subtilis* supplementation group; BSL: 1.50 g/kg *Bacillus subtilis* supplementation group; BSM: 3.00 g/kg *Bacillus subtilis* supplementation group; BSH: 4.50 g/kg *Bacillus subtilis* supplementation group.

**Figure 2:**
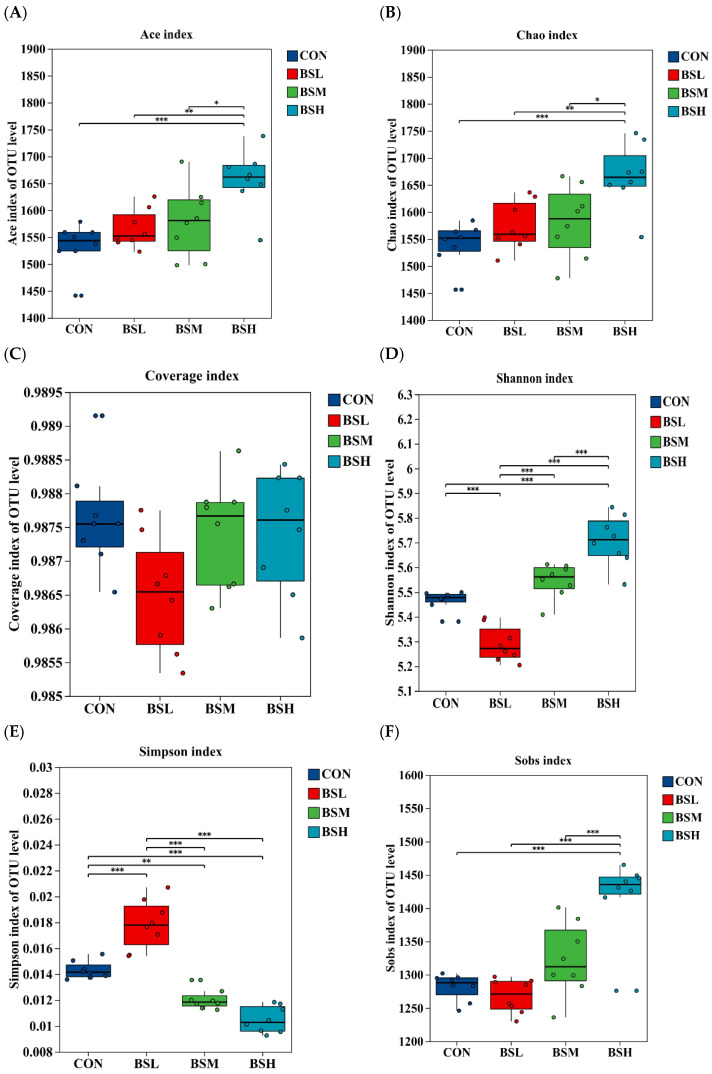
Alpha diversity index at OTU levels in rumen bacteria at 48 h incubation time across the four treatment groups (*n* = 8 per treatment). (**A**), ace index; (**B**) Chao index; (**C**) Coverage index; (**D**) Shannon index; (**E**) Simpson index; (**F**) Sobs index. CON: no *Bacillus subtilis* supplementation group; BSL: 1.50 g/kg *Bacillus subtilis* supplementation group; BSM: 3.00 g/kg *Bacillus subtilis* supplementation group; BSH: 4.50 g/kg *Bacillus subtilis* supplementation group. * *p* < 0.05; ** *p* < 0.01; *** *p* < 0.001.

In terms of community composition, 3, 4, 1, and 16 OTUs were unique to the CON, BSL, BSM, and BSH groups, respectively, and a total of 1586 OTUs were shared among the four groups (Figure 1). The Ace index, Chao index, and Sobs index increased with increasing BS supplementation levels (*p* < 0.05; Figure 2A,B,F). In addition, the Shannon index was lowest in the BSL group and highest in the BSH group (*p* < 0.05; Figure 2D). In contrast, the Simpson index was highest in the BSL group and lowest in the BSH group (*p* < 0.05; Figure 2F). There was no difference in the coverage index among the four groups (*p* > 0.10, Figure 2C).

**Figure 3:**
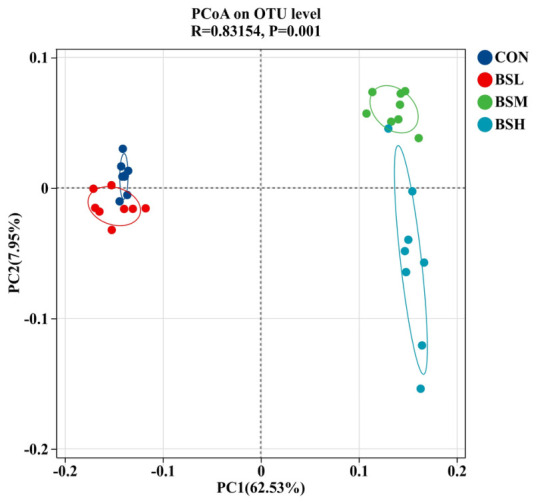
PCoA analysis based on Bray–Curtis distance in rumen bacteria at 48 h incubation time across the four treatment groups (*n* = 8 per treatment). CON: no *Bacillus subtilis* supplementation group; BSL: 1.50 g/kg *Bacillus subtilis* supplementation group; BSM: 3.00 g/kg *Bacillus subtilis* supplementation group; BSH: 4.50 g/kg *Bacillus subtilis* supplementation group.

**Figure 4:**
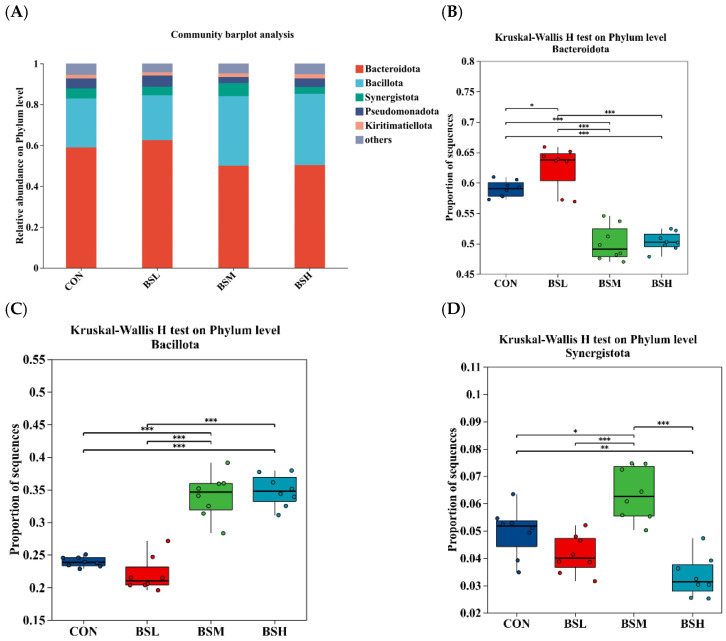

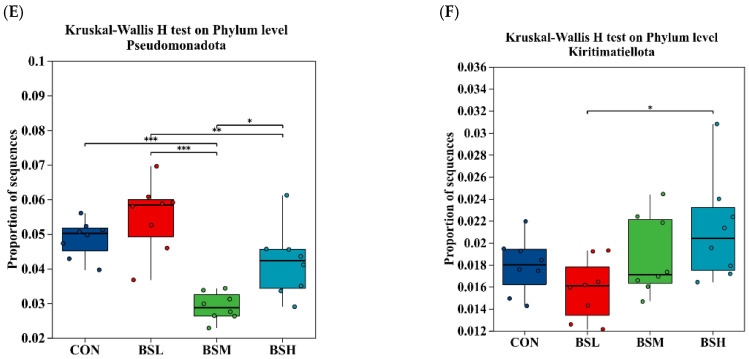
Relative abundance of the top five phylum at 48 h incubation time across the four treatment groups (*n* = 8 per treatment). (**A**) the relative abundance at the phylum levels; (**B**) the relative abundance of Bacteroidota; (**C**) the relative abundance of Bacillota; (**D**) the relative abundance of Synergistota; (**E**) the relative abundance of Pseudomonadota; (**F**) the relative abundance of Kirtimatiellota. CON: no *Bacillus subtilis* supplementation group; BSL: 1.50 g/kg *Bacillus subtilis* supplementation group; BSM: 3.00 g/kg *Bacillus subtilis* supplementation group; BSH: 4.50 g/kg *Bacillus subtilis* supplementation group. * *p* < 0.05; ** *p* < 0.01; *** *p* < 0.001.

To examine the disparities in the rumen microbial community between the two groups, a beta-diversity analysis was conducted (Figure 3). According to the PCoA plots derived from the Bray–Curtis distance matrix, the points representing rumen microorganisms among the four groups were clearly separated on the coordinate axis. A total of 33 bacterial phyla and 365 genera were identified from the 32 fermentation fluid samples. Bacteroidota, Bacillota, and Synergistota were the dominant phyla in the fermentation fluid samples, accounting for 55.49 %, 28.70 %, and 4.70 %, respectively (Figure 4A). The relative abundance of Bacteroidota and Pseudomonadota were highest in the BSL group and lowest in the BSM group (*p* < 0.05; Figure 4B,E), whereas Bacillota and Kiritimatiellota were highest in the BSH group and lowest in the BSL group (*p* < 0.05; Figure 4C,F). Synergistota was highest in the BSM group and lowest in the BSH group (*p* < 0.05; Figure 4D).

**Figure 5:**
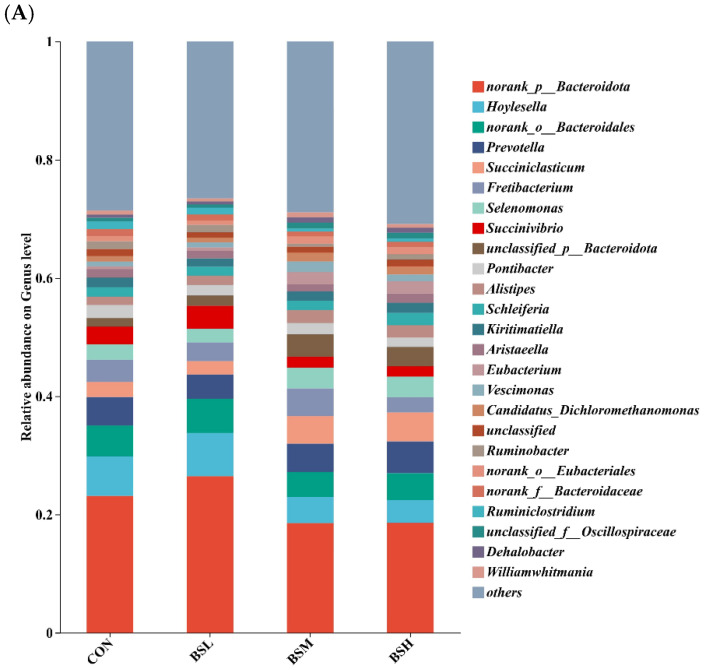

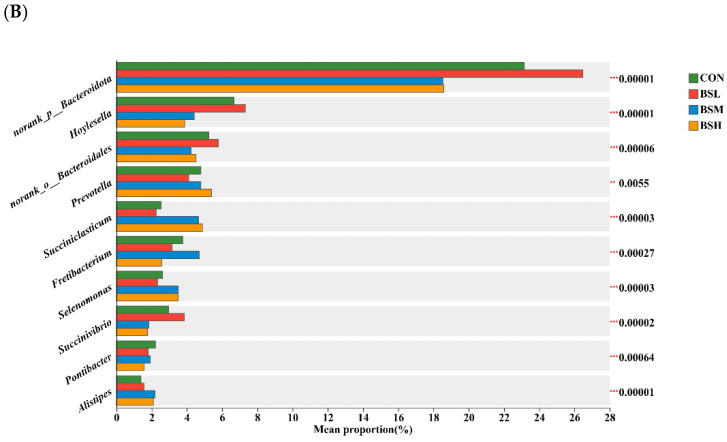
Bacterial relative abundances of the top 25 genera at 48 h incubation time across the four treatment groups (*n* = 8 per treatment). (**A**) the relative abundance at the genus levels; (**B**) the difference genus among the 4 groups. CON: no *Bacillus subtilis* supplementation group; BSL: 1.50 g/kg *Bacillus subtilis* supplementation group; BSM: 3.00 g/kg *Bacillus subtilis* supplementation group; BSH: 4.50 g/kg *Bacillus subtilis* supplementation group. ** *p* < 0.01; *** *p* < 0.001.

At the genus levels, the most dominant was *norank_p_Bacteroidota*, followed by *Hoylesella*, *norank_o_Bacteroidoles*, *Prevotella*, *Succiniclsaticum*, and *Fretibacterium* (Figure 5A). The relative abundances of *norank_p_Bacteroidota* and *norank_o_Bacteroidoles* were highest in the BSL group, whereas they were lowest in the BSM group (*p* < 0.05; Figure 5B). The relative abundances *Hoyesella* and *Succinivibrio* were highest in the BSL group, whereas they were lowest in the BSH group (*p* < 0.05). The relative abundances of *Prevotella*, *Succiniclasticum*, and *Selenomonas* were highest in the BSM group, whereas they were lowest in the BSL group (*p* < 0.05).

**Figure 6:**
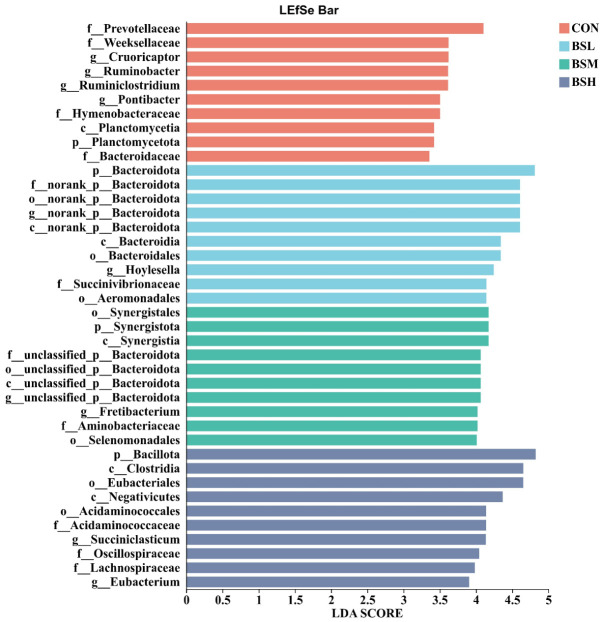
Linear discriminant analysis effect size (LEfSe) analysis displaying the differential species-characteristic taxon with a threshold value of 2. Prefixes represent abbreviations for the taxonomic rank of each taxon, class (c^−^), order (o^−^), family (f^−^) and genus (g^−^). CON: no *Bacillus subtilis* supplementation group; BSL: 1.50 g/kg *Bacillus subtilis* supplementation group; BSM: 3.00 g/kg *Bacillus subtilis* supplementation group; BSH: 4.50 g/kg *Bacillus subtilis* supplementation group.

The variations in the bacterial communities among the four BS supplementation groups were further explored using linear discriminant analysis effect size (LEfSe) analysis (Figure 6). At the genus levels, *Cruoricaptor*, *Ruminiclostridium*, and *Pontibacter* were biomarkers in the CON group; *norank_p_Bacteroidota* and *Hoylesella* were biomarkers in the BSL group; *unclassified_p_Bacteroidota* and *Fretibacterium* were biomarkers in the BSM group; and *Succiniclasticum* and *Eubacterium* were biomarkers in the BSH group.

### 3.4. Correlation Analysis Between Ruminal Microbiota and Fermentation Traits

**Figure 7:**
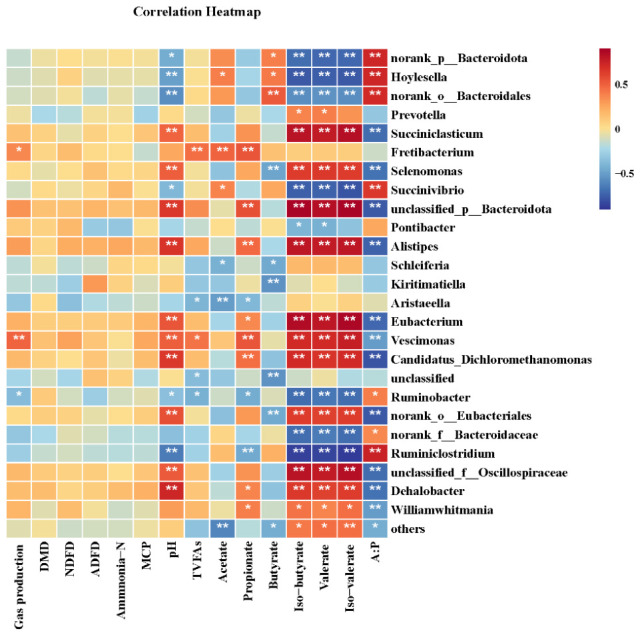
Pearson correlations between gas production, in vitro nutrient degradability, fermentation parameters and rumen bacteria of the top 25 genera. DMD: dry matter degradability; NDFD: neutral detergent fiber degradability; ADFD: acid detergent fiber degradability; MCP: microbial protein; TVFAs: total volatile fatty acids; A:P: the ratio of acetate to propionate. * *p* < 0.05; ** *p* < 0.01.

To investigate the interplay between rumen microbial communities and fermentation parameters, a Pearson correlation was undertaken to examine the relationship between gas production, in vitro nutrient degradability, fermentation parameters and rumen bacteria in the top 25 genera (Figure 7). A total of 130 correlations was found, which consisted of 73 positive correlations and 57 negative correlations.

*norank_p_Bacteroidota* and *norank_o_Bacteroidoles* were positively correlated with butyrate and the ratio of acetate to propionate, and negatively correlated with pH, iso-butyrate, valerate, and iso-valerate. *Hoylesella* was positively correlated with acetate and butyrate, and negatively correlated with pH, iso-butyrate, valerate, and iso-valerate. *Succiniclasticum* was positively correlated with pH, iso-butyrate, valerate, and iso-valerate, and negatively correlated with the ratio of acetate to propionate. *unclassified_p_Bacteroidota*, *Alistipes*, *Eubacterium*, *Candidatus_Dichloro-methanomonas*, and *Dehalobacter* were positively correlated with pH, propionate, iso-butyrate, valerate, and iso-valerate, and negatively correlated with the ratio of acetate to propionate.

## 4. Discussion

### 4.1. The Effect of Different Bacillus subtilis Supplementation Levels on In Vitro Gas Production and In Vitro Nutrient Degradability

The in vitro gas production technique was used to evaluate the fermentation traits of the *Macadamia integrifolia* husk, as the volume of gas produced is positively related to the amount of nutrients fermented. Commonly, probiotics can increase rumen fermentation and nutrient digestion by altering rumen functions and reducing undesirable microbes, as previously reported [23,24]. Gas production increased as excepted when *Bacillus subtilis* was added to the MIH, which is in agreement with a previous in vitro study of new multistrain *Bacilli*, *Lactobacilli*, yeast, and their mixtures in sheep [25]. The previous study reported that supplementation with a *Bacillus*-based direct-fed microbial probiotic could improve the dry matter digestibility and neutral detergent fiber digestibility at the 24 h and 48 h incubation times [26]. In addition, a 1% ratio of *Lactobacillus plantarum* and *Saccharomyces cerevisiae* added to a fermented rice straw-based diet could increase the digestibilities of dry matter, neutral detergent fiber, and acid detergent fiber [27]. In the present study, we found that the digestibilities of dry matter, neutral detergent fiber, and acid detergent fiber were increased in *Bacillus subtilis* supplementation groups and highest in BSL and BSM groups. This can be explained because the probiotic can digest the fiber components [28].

### 4.2. The Effect of Different Bacillus subtilis Supplementation Levels on In Vitro Rumen Fermentation Parameters

A stable ruminal pH, which ranged from 6.2 to 7.2, is vital for rumen fermentation and function in ruminants [29]. In the present study, the ruminal pH values were between 6.48 and 6.55; all of these values were within the normal ranges. In addition, the ruminal pH was not affected by *Bacillus subtilis* supplementation, which is in agreement with a previous in vitro study [30] and in vivo studies [31,32]. In the present study, a higher ammonia-N concentration was seen in the *Bacillus subtilis* supplementation groups. This was possibly related to an increased crude protein digestibility in ruminants when supplemented with *Bacillus subtilis* [33] or symbiotic lignocellulose-degrading bacteria [34]. Unfortunately, the crude protein degradability was not measured, and this index needs to be considered in future studies. In ruminants, MCP provides 60–85% of the total amino acids for the small intestine in ruminants [35]. Previous studies reported that probiotics, for example, could improve the MCP concentration [36,37]. The increased MCP concentration in the *Bacillus subtilis* groups could be explained by higher nutrients for microbial utilization and the superior activity of beneficial rumen bacteria, which outcompete undesirable species and improve ammonia utilization to synthesize MCP [38].

The inclusion of a probiotic in the diet influenced key ruminal fermentation parameters, particularly total VFA concentrations and the overall VFA profile [39]. A previous in vitro study reported that the concentrations of acetate, propionate, and butyrate were improved when 10^9^ CFU live *Bacillus subtilis* was added to natto [40]. In the present study, we found that the concentrations of acetate, propionate, and butyrate were increased in the BS supplementation groups compared to the CON group, which is partly in agreement with a previous in vitro study [40]. This shift, along with the rise in total VFA concentration, especially in the BSL and BSM groups, suggested that 1.5 g/kg and 3.0 g/kg BS supplementation may promote more favorable fermentation conditions, for example, improving the total VFA concentration. The ratio of acetate to propionate is affected by numerous factors and is determined by the fermentation type [41]. For a low-concentrate diet, rumen fermentation occurs mainly as the acetate type, whereas, for a high-concentrate diet, rumen fermentation occurs mainly as the propionate type. A previous in vitro study showed that 10^9^ CFU live *Bacillus subtilis* added to natto did not affect the ratio of acetate to propionate when used in multiparous lactating Holstein cows as rumen fluid donors [40]. In contrast, we found that the ratio of acetate to propionate was altered among the four groups. This difference could be explained by the BS supplementation levels.

### 4.3. The Effect of Different Bacillus subtilis Supplementation Levels on In Vitro Rumen Bacterial Communities

The rumen microbial community maintains a delicate equilibrium, ensuring rumen homeostasis and optimizing nutrient digestion. In Guiqian semi-fine wool lambs, supplementation with probiotics altered the rumen microbial architecture, with the 2000 mg/kg group demonstrating marked differences in both α-diversity and β-diversity compared with the CON group [42]. Moreover, in Hu sheep, the addition of BS significantly improved Chao1 and ACE indices in the colon and cecum compared to the control group [15]. In the present study, we found that the alpha diversity was changes by BS supplementation, which suggested a more diverse and complex microbial composition.

In the present study, Bacillota and Bacteroidota emerged as the dominant phyla among the four BS supplementation groups, collectively representing 84.2% of the rumen microbiota. These results are in agreement with previous in vitro studies reported in goats [43] and sheep [31], and in vivo studies reported in Hu sheep [38] and in Guiqian semi-fine wool sheep [42]. A previous study reported that the relative abundance of Bacillota was greater and the relative abundance of Bacteroidetes was lesser in probiotic compound groups than in the control group in the rumen fluid of Hu sheep [38]. In addition, supplementation with a 2% probiotic blend exhibited increased Bacillota and reduced Bacteroidota in Guiqian semi-fine wool lambs [42]. However, the relative abundance of Bacillota and Bacteroidota increased in Angus cattle when offered fungal probiotics [44] and in colon of Hu lambs who consumed *Bacillus subtilis* [15]. In the present study, we found that the relative abundance of Bacteroidota was greatest and Bacillota was lowest in the BSL group. The different results among these studies could be explained by the probiotic sources and their supplementation levels. Synergistota could ferment amino acids for energy and potentially detoxify harmful compounds in plants eaten by grazed ruminants [45]. In the present study, we found that the relative abundance of Synergistota was lowest in the BSL group, which could explain the increased MCP concentration in the BSL group.

Previous in vitro studies reported that the dominant genera were *Escherichia-Shigella* and *Streptococcus* in Taihang White cashmere goats [46], *Prevotella 1* in Crossbreed Boer female goats [47], *Lactobacillus* and *Acetobacter* in lactating Holstein cows [47], and Fibrobacter in Holstein cows [48]. In addition, in in vivo studies, the dominant genera were *norank_f_082* and *Prevotella* in Dazu black kids [49], *Prevotella* and *Ruminococcus* in Hu sheep [45], and *Prevotella* and *Rikenellaceae_RC9_gut_group* in Boer goat × Shandong local white goat [50]. In the present study, we found that the dominant genera were *norank_p_Bacteroidota* and *Hoylesella*. This difference among the dominant bacterial communities could be explained by the dietary nutritional composition and animal species and their ages. *Fretibacterium* is an anaerobic genus that belongs to the Synergistetes phylum. It was reported that *Fretibacterium* was significantly increased by *B. subtilis* 810 in an in vitro study of Holsteins cows [51]. In the present study, we found the *Fretibacterium* was highest in the BSM group and lowest in the BSH group among the four groups. In addition, the relative abundance of *Fretibacterium* was reportedly increased in moderate grazing with 2% concentrate supplementation in weaned Hulunbuir male lambs [52] and in highest-milk-fat-content buffaloes [53]. In the present study, we found that *Fretibacterium* was positively associated with total volatile fatty acids, acetate, and propionate. *Succinivibrio* refers to a genus that produces succinic acid, and was highest in the BSL group; however, *Succinniclassicum* can utilize succinate to produce propionate [54], and the relative abundance of this genus was highest in the BSM group among the four groups. This could partly explain why the concentration of propionate was greater in the BSL and BSM groups. In addition, our results indicated that the relative abundance of *Succiniclasticum* was positively correlated with the concentration of valerate, which is in agreement with a previous study [55]. In contrast, we found the *Succinivibrio* was negatively correlated with the concentration of valerate.

## 5. Conclusions

*Macadamia integrifolia* husk supplemented with *Bacillus subtilis* could improve gas production, dry matter disappearance, neutral and acid detergent fiber disappearance, and rumen fermentation traits. Moreover, the bacterial communities were altered; for example, the relative abundances of *norank_p_Bacteroidota*, *norank_o_Bacteroidoles*, *Prevotella*, *Succiniclasticum*, and *Selenomonas* were changed with *Bacillus subtilis* supplementation levels. In conclusion, the supplementation of 1.5 to 3.0 g/kg *Bacillus subtilis* on *Macadamia integrifolia* husk could improve its utilization as a feasible alternative feedstuff in ruminants using an in vitro approach. Further in vivo studies are needed to evaluate growth performance when using MIH as a feedstuff.

## Figures and Tables

**Table 1:** Gas production (mL/0.4 g DM) in response to different BS inclusion levels in rumen inocula of goats (*n* = 8 per treatment).

Items	CON	BSL	BSM	BSH	SEM	*p*-Values		
						BS	BS-L	BS-Q
3 h	10.2	10.3	11.1	10.2	0.18	0.028	0.621	0.077
6 h	16.9	18.4	17.5	16.8	0.22	0.030	0.422	0.009
9 h	20.9	21.5	21.9	21.4	0.23	0.043	0.778	0.080
12 h	25.1	26.3	26.8	25.7	0.45	0.027	0.858	0.083
24 h	34.6	35.8	36.7	35.6	0.50	0.045	0.678	0.150
48 h	44.1	45.3	47.1	44.0	0.41	0.022	0.488	0.012

CON: no *Bacillus subtilis* supplementation group; BSL: 1.50 g/kg *Bacillus subtilis* supplementation group; BSM: 3.00 g/kg *Bacillus subtilis* supplementation group; BSH: 4.50 g/kg *Bacillus subtilis* supplementation group. BS: the effect of *Bacillus subtilis*; BS-L: the linear effect of *Bacillus subtilis*; BS-Q: the quadratic effect of *Bacillus subtilis*.

**Table 2:** In vitro nutrient degradability in response to different BS inclusion levels in rumen inocula of goats (*n* = 8 per treatment).

Items	Time	CON	BSL	BSM	BSH	SEM	*p*-Values		
							BS	BS-L	BS-Q
DMD, g/kg	6	257	269	274	263	3.0	0.020	0.340	0.062
	12	333	344	351	357	3.8	0.031	0.023	0.702
	24	415	445	447	410	6.0	0.038	0.808	0.005
	48	484	501	494	499	4.9	0.049	0.344	0.026
NDFD, g/kg	6	219	237	249	243	5.0	0.040	0.416	0.766
	12	306	313	326	310	2.3	0.027	0.616	0.313
	24	370	375	392	372	3.0	0.027	0.923	0.52
	48	402	426	436	402	3.4	0.016	0.163	0.664
ADFD, g/kg	6	134	142	148	128	2.3	0.026	0.745	0.118
	12	213	228	235	223	3.0	0.035	0.521	0.297
	24	271	294	309	282	3.9	0.047	0.618	0.285
	48	315	352	368	325	4.4	0.014	0.258	0.755

CON: no *Bacillus subtilis* supplementation group; BSL: 1.50 g/kg *Bacillus subtilis* supplementation group; BSM: 3.00 g/kg *Bacillus subtilis* supplementation group; BSH: 4.50 g/kg *Bacillus subtilis* supplementation group; DMD, dry matter degradability; NDFD, neutral detergent fiber degradability; ADFD, acid detergent fiber degradability. BS: the effect of *Bacillus subtilis*; BS-L: the linear effect of *Bacillus subtilis*; BS-Q: the quadratic effect of *Bacillus subtilis*.

**Table 3:** The fermentation parameters at 48 h incubation time in response to different BS inclusion levels in rumen inocula of goats (*n* = 8 per treatment).

Items	CON	BSL	BSM	BSH	SEM	*p*-Values		
						BS	BS-L	BS-Q
pH	6.48	6.50	6.55	6.53	0.017	0.201	0.301	0.305
Ammonia-N, mg/100 mL	5.113	5.756	5.557	5.528	0.077	0.019	0.099	0.021
MCP, mg/100 mL	14.154	15.114	15.009	14.964	0.271	0.026	0.071	0.038
Total VFAs, mmol/L	27.58	29.11	30.16	27.53	0.242	<0.001	0.493	<0.001
Acetate, mmol/L	19.098	20.075	20.028	18.366	0.148	<0.001	0.004	<0.001
Propionate, mmol/L	4.806	5.025	5.326	4.906	0.044	<0.001	0.020	<0.001
Butyrate, mmol/L	0.406	0.442	0.639	0.591	0.020	0.01	0.06	0.00
Iso-butyrate, mmol/L	2.272	2.479	2.383	2.084	0.044	<0.001	<0.001	0.026
Valerate, mmol/L	0.699	0.767	1.285	1.140	0.049	<0.001	<0.001	0.027
Iso-valerate, mmol/L	0.299	0.318	0.503	0.443	0.017	<0.001	<0.001	0.019
Acetate: Propionate	3.98	4.00	3.76	3.75	0.03	<0.001	<0.001	0.62

CON: no *Bacillus subtilis* supplementation group; BSL: 1.50 g/kg *Bacillus subtilis* supplementation group; BSM: 3.00 g/kg *Bacillus subtilis* supplementation group; BSH: 4.50 g/kg *Bacillus subtilis* supplementation group. MCP, microbial protein. BS: the effect of *Bacillus subtilis*; BS-L: the linear effect of *Bacillus subtilis*; BS-Q: the quadratic effect of *Bacillus subtilis*.

## Data Availability

The original contributions presented in this study are included in the article. Further inquiries can be directed to the corresponding authors.
